# Development of graphene-modified jute/glass hybrid composites *via* fiber wrapping for enhanced structural applications

**DOI:** 10.1039/d5ra02968f

**Published:** 2025-08-04

**Authors:** Mainul Islam, Md Tarikul Islam, Main Uddin Apu, Ariful Islam, Shahjalal Khandaker, Forkan Sarker

**Affiliations:** a Department of Textile Engineering, Dhaka University of Engineering and Technology Gazipur 1707 Bangladesh forkan@duet.ac.bd

## Abstract

Jute fiber, an abundantly available natural fiber, is increasingly explored for structural composite applications. However, its inherent flaws and the limited development of optimized textile architectures have restricted its use in high load-bearing engineering applications. Hybridization with glass fiber offers a promising route to enhance the mechanical performance of jute composites while reducing the reliance on synthetic materials. Nonetheless, challenges remain in achieving high jute content and sufficient strength within hybrid structures. To address these limitations, this study presents the development of a novel hybrid preform architecture using a fiber wrapping technique, where glass fibers serve as the core and jute fibers are wrapped around them. Additionally, graphene oxide (GO) treatment was applied to modify jute fibers, improving their compatibility with glass fibers. Mechanical testing revealed that the GO-treated hybrid composite (G.cGFJF) demonstrated the highest performance, achieving a tensile strength of 272.63 MPa, a flexural modulus of 10.42 GPa, and an impact resistance of 85.56 kJ m^−2^. Moreover, water absorption was significantly reduced to 1.12% in GO-coated samples, attributed to enhanced surface hydrophobicity and interfacial bonding. These results highlight the strong synergistic effects between the high-strength glass fiber core and the interlocking jute fiber wrap, indicating that GO-modified jute/glass hybrid composites hold great promise for next-generation structural components in automotive, aerospace, and civil engineering sectors where performance and sustainability are critical.

## Abstract 2

Jute fiber, an abundantly available natural fiber, is increasingly explored for structural composite applications.

## Graphical abstract

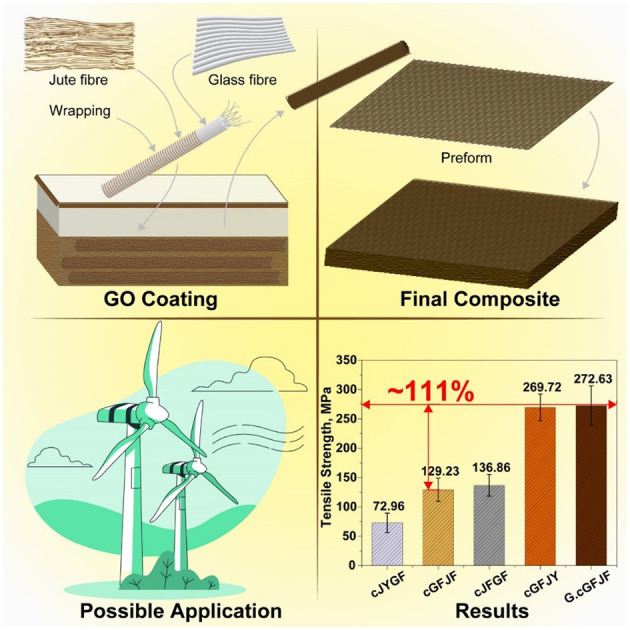

## 1. Introduction

In many structural applications, lightweight and high-specific-strength fibre-reinforced composites are becoming increasingly popular. Recent technological advancements in developing new materials, improving manufacturing techniques and modifying existing materials play a vital role in replacing traditional materials.^1,2^ In fibre-reinforced composites, synthetic materials such as glass fibre, carbon fibre and Kevlar fibre combined with thermoplastic or thermoset matrices, dominate various structural applications.^3^ Among them, glass fibre accounts for more than 60% of synthetic fibre use in composite applications. Major application areas of glass fibre-based composites include the automotive industry, aerospace, sports, leisure boats, and furniture^4,5^ However, in applications where high load-bearing capacity is not a critical requirement, natural fibre reinforcements such as jute, flax, hemp, and sisal can serve as viable alternatives due to their comparable specific properties. In particular, long-fibre jute has gained significant attention in recent years compared to other plant-based fibres. This is due to its abundance, ease of cultivation, lower cost, and mechanical properties that are comparable to glass fibre.^6^

However, the reliability of using jute fibre as a replacement for glass fibre primarily depends on fibre preparation techniques, novel fibre architectures, preforming methods, and surface modifications. One technique for strengthening jute fibre was explained by Sarker^7^*et al.*, where they cleaned field-retted fibre, manually hackled it, and converted the unidirectional fibre into a dry fibre preform using a light compression technique. They claimed that in a vacuum resin infusion process, the fibre volume fraction of untreated jute fibre can be improved by more than 100%, significantly enhancing the mechanical properties of the composites, achieving a stiffness of 38 GPa and a strength of approximately 370 MPa. Building on this laboratory technique, Hasan *et al.*^8^ developed unidirectional mats from low-processed industrial slivers and converted them into UD sheets using binder and stitching techniques. Their composites achieved stiffness and strength values of approximately 16 GPa and 176 MPa, respectively, despite a fibre volume fraction of nearly 50%. To avoid damage caused by stitching and to enable scalable production of jute fibre-based UD preforms, Yeasin *et al.*^9^ proposed a non-crimp unidirectional jute fibre preform incorporating a small percentage of glass fibre in the weft direction. They claimed that this drapeable UD jute yarn preform is suitable for structural applications. Similarly, Shah *et al.*,^10^ using drum winding techniques, achieved maximum stiffness and strength values of 15 GPa and 155 MPa, respectively, in UD jute yarn composites, making them suitable for turbine blade applications. In a recent study, Ahamed^11,12^*et al.* reported that the individualization of field-retted jute fibres, followed by chemical modification, can further improve the tensile properties of composites, even when the fibres are arranged in a chopped form. Their study showed that highly cleaned and individualized fibres, when chopped to a minimum length of 5 mm, can enhance composite stiffness and strength to 5.5 GPa and 90 MPa, respectively. Additionally, they observed that the bending modulus and strength could reach 12 GPa and 186 MPa, respectively, due to the fibres' higher packing ability after chemical and physical modifications. Inspired by the concept of fibre individualization, fibre alignment in a parallel direction, and reduced fibre twist, the development of a novel jute fibre composite could be further explored after rigorous evaluation. Therefore, this study aims to utilize field-retted and highly individualized fibres to manufacture a new type of dry fibre preform from jute fibre.

Further strengthening of this novel preform through chemical modification using nanomaterials is in high demand in literature.^13–16^ However, since chemical modification of fibres can be hazardous and relatively costly, an alternative approach is hybridizing jute and glass fibres. This technique could be a key area of interest for researchers aiming to minimize glass fibre content while maximizing jute fibre content in preforms. Therefore, modification of jute fibres using widely explored graphene oxide will be used in this study which is influenced from our previous work.^7^

In hybridization techniques, woven jute and glass fibres are typically used, with studies focusing on the effects of layer sequences and placement techniques, as documented in the literature.^17–21^ In this case, the maximum tensile strength of the composites (∼110 MPa) was reported by Sezgin *et al.*,^22^ where glass and jute fibres were placed in equal balanced layers and aligned in the warp direction of the composite. However, when jute and glass fibres were interwoven or interplay arranged during the weaving process of the preform, the tensile strength of the composite was further reduced to ∼50 MPa, as reported by Ouarhim *et al.*^23^ These studies in the literature support the use of hybrid composites in strength-demanding applications. However, their limitations in architectural development—such as high crimp, inadequate resin impregnation, and increased stress concentrations—may affect their performance. To address these challenges, the wrapping technique, which is widely used for reinforcing fibres and thermoplastic matrices, can be employed. This technique allows for better fibre distribution and improved impregnation, followed by consolidation into composites using compression molding.^24–27^

In this technique, two fibres are hybridized either by mixing them together during the spinning process (wrap spinning) or by twisting two types of rovings or filaments.^25,28^ Finally, they are converted into woven or unidirectional (UD) architectures to manufacture dry fibre preforms suitable for composite applications. However, the wrapping of two reinforcing fibres, such as jute and glass, is still limited in the literature. No studies have yet claimed the development of a hybrid jute–glass fibre preform using wrapping techniques.

In this study, we report for the first time the use of highly individualized field-retted jute fibre rovings in a wrapping process, where jute fibres are placed in the core and glass fibres serve as the wrapping material. Additionally, this study examines the performance of hybrid jute–glass fibre preforms when the arrangement is reversed—placing glass fibres in the core and jute fibres as the wrapping material. To maximize the mechanical properties of the composites, these novel preforms were manufactured and aligned in the zero-degree direction to fabricate unidirectional (UD) jute–glass hybrid composites. Tensile, bending, and impact properties were evaluated to assess the mechanical performance of the composites, while water absorption studies were conducted to examine their aging behavior in water.

## 2. Experimental methods

### 2.1 Materials

Jute fibre and glass fibre were selected as the primary materials for the experimental process. Jute fibre was collected from local market in Bangladesh. Glass fibre, epoxy resin,^29,30^ and amine hardener were purchased from Lucky Fibre and Acrylic, Dhaka, Bangladesh. Graphite powder was collected from the Bangladesh Atomic Energy Commission. The chemicals used in the study, including sulfuric acid (H_2_SO_4_, 98%), sodium nitrate (NaNO_3_), potassium permanganate (KMnO_4_), hydrochloric acid (HCl), hydrogen peroxide (H_2_O_2_, 30%), ethanol, and filter paper, were procured from Modern Scientific, Dhaka, Bangladesh. The steel frame used in the experiments was made at a local workshop.

### 2.2 Graphene oxide synthesis process

**Fig. 1:**
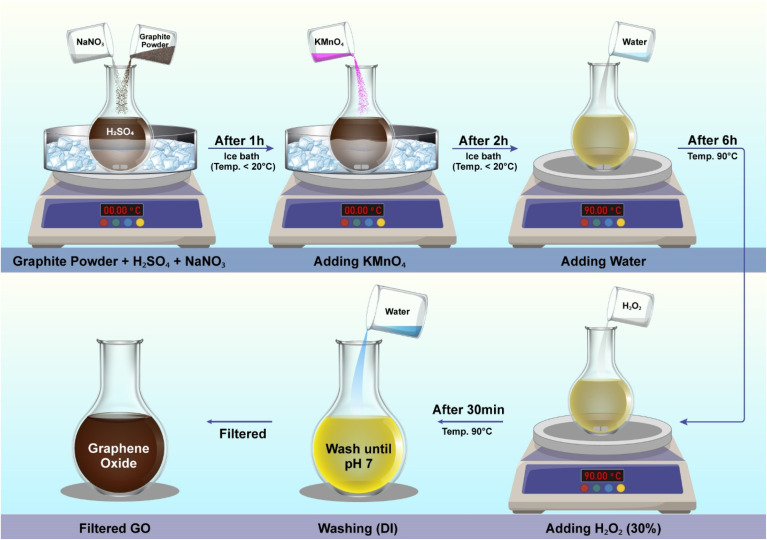
Graphene oxide synthesis process.

The synthesis of graphene oxide (GO) using the Modified Hummers Method that transforms graphite into an oxygen-rich material.^31^ Graphene oxide synthesis process shown in Fig. 1. First of all, graphite powder was added to concentrated sulfuric acid (H_2_SO_4_, ∼98%) in a reaction vessel in 1 g to 23 mL ratio, along with 0.5 g to 1 g NaNO_3_ to graphite powder. This mixture was stirred thoroughly to ensure uniform dispersion and was left to react for one hour. The sulfuric acid played a crucial role to introduce oxygen groups to the graphite layers. Next, KMnO_4_ (graphite : KMnO_4_ = 3 : 1) was slowly introduced into the reaction mixture while keeping the temperature below 20 °C using an ice bath. This step requires careful and gradual addition to prevent excessive heat generation. The potassium permanganate acts as a strong oxidizing agent, initiating the oxidation of graphite and the formation of oxygen-containing functional groups. After two hours, the ice bath was removed and the temperature was gradually raised to 35 °C. The mixture was continued the stirring for six hours for further oxidation. Once oxidation was completed, 50 mL of distilled water for per 1 g graphite powder was added slowly to the reaction vessel. The temperature was increased to 90 °C and maintained for 30 minutes to promote complete oxidation and breakdown of any unreacted graphite particles. To neutralize any remaining oxidizing agents, 150 mL of distilled water for per 1 g graphite powder mixed with 10% of water hydrogen peroxide (H_2_O_2_ ∼ 30%) is added to the reaction mixture. The hydrogen peroxide reduces residual permanganate and manganese dioxide into soluble manganese ions. This reaction results in a noticeable color change, turning the solution from dark brown to a lighter yellow shade. It confirming the complete oxidation process. The final stage involved the purification of graphene oxide by washing the mixture sequentially with 5% HCl, ethanol, and distilled water multiple times. This washing process is repeated until the pH of the solution reaches neutral (pH 7). After filtration, the graphene oxide suspension was obtained.

### 2.3 Glass fibre and jute fibre/yarn wrapping process

**Fig. 2:**
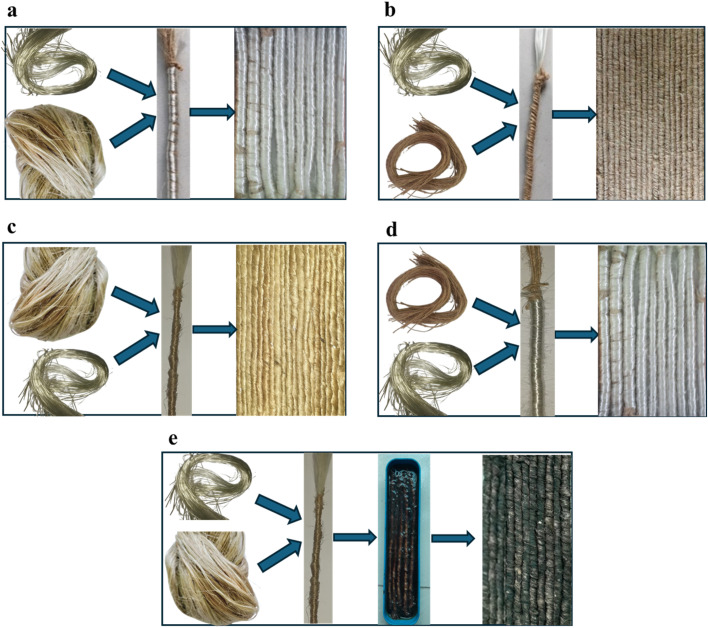
Development hybrid wrapped roving: (a) raw jute in the core wrapped by glass fibre; (b) glass fibre in the core wrapped by commercial jute fibre; (c) glass fibre in the core wrapped by field retted jute fibre; (d) commercial yarn in the core wrapped by glass fibre and (e) dip coating of hybrid roving in the GO solution.

The jute fibres were individualized manually using a hand combing technique to separate the technical fibres. The glass fibres were handled carefully to avoid breakage and maintain uniformity in wrapping. The wrapping process was carried out in four different stages to create different core-fibre arrangements (Fig. 2). In the first configuration, a bundle of glass fibre was used as the core, with jute fibre wrapped around it to form the outer layer. The second configuration involved a bundle of jute fibre as the core, wrapped with glass fibres. In the third configuration, a bundle of glass fibre was used as the core and jute yarn wrapped around it. Lastly, in the fourth configuration, a bundle of jute yarn served as the core, with glass fibres wrapped around it. All jute glass wrapped stands cut into 20 cm.

### 2.4 Coating process by graphene oxide

The first wrapping process (glass fibre was used as the core, with jute fibre wrapped around it) used for GO coating. To achieve uniform GO deposition, the hybrid fibre strands were immersed in an aqueous GO suspension with a concentration of 1 wt%.^32^ The immersion process was carried out at ambient room temperature for a duration of 30 minutes to facilitate sufficient adsorption of GO onto the fibre surfaces through non-covalent interactions. It is including hydrogen bonding and van der Waals forces. Following the dipping process the strands were removed and subjected to thermal drying at 80 °C for 2 hours to eliminate moisture and ensure proper adhesion of the GO coating. The drying step was critical for enhancing coating stability and promoting partial reduction of GO which can improve electrical and mechanical properties. The coated strands were allowed to cure at room temperature for 24 hours to ensure complete stabilization of the GO layer on the fibre surface. This procedure ensured a uniform and adherent GO coating suitable for further composite fabrication.

### 2.5 Composite preparation

**Fig. 3:**
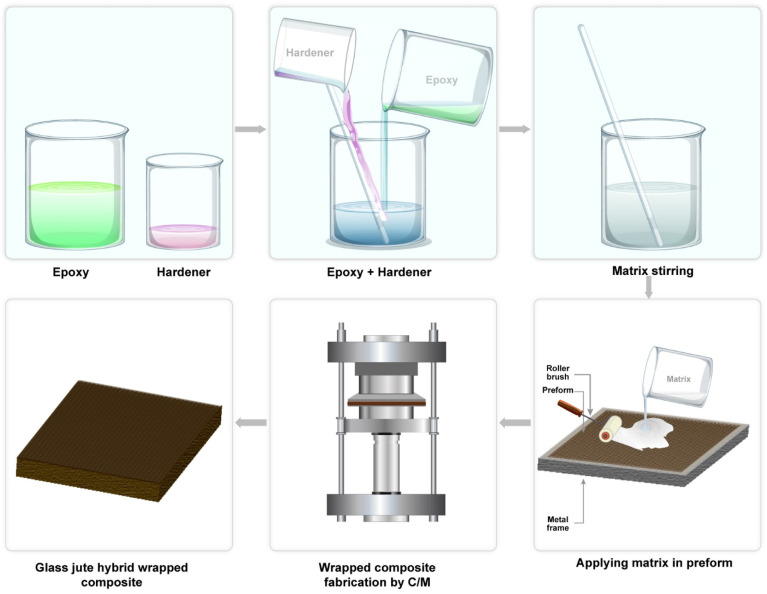
Composite fabrication process.

The hybrid composites were fabricated using a still-frame setup. The epoxy resin is used as a matrix material. Four types of wrapped and graphene oxide (GO)-coated glass–jute stands were used for preparing five composite samples (Table 1). Initially, the stands were arranged longitudinally in a fixed frame (200 mm × 150 mm) under tension ensuring uniform alignment (Fig. 3). The epoxy matrix was prepared by mixing epoxy resin and amine hardener in a 3 : 1 ratio. The mixture was stirred gently to achieve homogeneity. Once the resin mixture was ready, it was carefully applied on the aligned glass–jute stands in frame using the hand lay-up technique to ensure thorough impregnation. Polytetrafluoroethylene (PTFE) sheets were placed on both the upper and lower surfaces of the frame structure to facilitate composites. During the compression molding process, the upper plate remained fixed while the lower plate was movable. The composites were subjected to a high compressive pressure of 2 ton per square inch at a temperature of 70 °C for 1 hour in the molding machine. After the curing process, an air-cooling system was employed to gradually reduce the temperature of the composites to room temperature. Once cooled, the samples were carefully removed from the mold and prepared for further processing. To ensure complete curing, the composites underwent a post-curing phase for 24 hours at room temperature.

**Table 1:** Different glass jute wrapped and go coated composites and their coding

No	Name	Code
01	Core jute yarn and wrapping by glass fibre	cJYGF
02	Core glass fibre and wrapping by jute yarn	cGFJY
03	Core jute fibre and wrapping by glass fibre	cJFGF
04	Core glass fibre and wrapping by jute fibre	cGFJF
05	GO treated core glass fibre and wrapping by jute fibre	G.cGFJF

## 3. Characterization

### 3.1 Tensile test

**Fig. 4:**
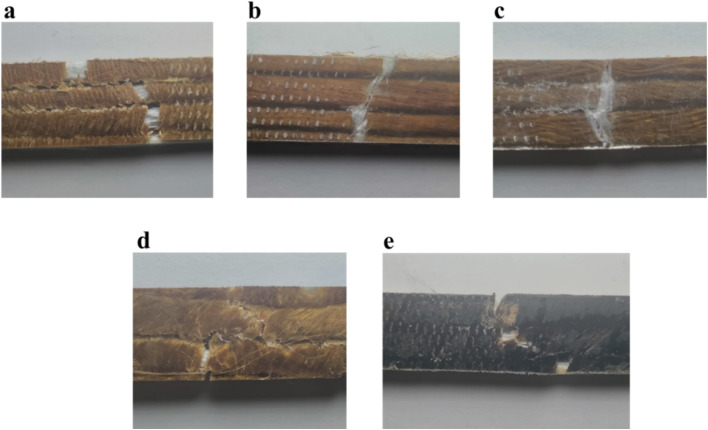
Samples after tensile test: (a) cGFJY, (b) cJFGF, (c) cJYGF, (d) cGFJF, (e) G.c cGFJF.

The tensile test was performed following ASTM D638 standards using a universal AG-X Plus testing machine.^33^ The test was conducted with a crosshead speed of 5 mm min^−1^ and a machine gauge length of 50 mm. The specimens, prepared according to the same standard, measured 165 mm × 15 mm × 3 mm. Each type of sample was tested in triplicate to ensure consistency (Fig. 4). The study analyzed both the average values and standard deviations of the obtained results.

### 3.2 Flexural test

Flexural testing was carried out at room temperature using a universal testing machine (UTM) configured for a three-point bending test in accordance with ASTM D790 standards.^34^ The test was conducted with a crosshead speed of 1.4 mm min^−1^ and a machine span length of 50 mm. The specimens had dimensions of 127.5 mm × 12.5 mm × 3 mm. Each sample type was tested using three composite specimens, and the results were analyzed accordingly.

### 3.3 Impact test

Impact testing of the composite samples was conducted following ASTM D256 standards. The specimens, measuring 80 mm × 10 mm × 3 mm, were positioned vertically within the testing apparatus.^35^ A pendulum weighing 2.634 kg was released from an elevated angle of 150° to strike the sample. The impact angle was displayed on the tester, and the corresponding impact energy was determined using the reference chart provided within the impact testing machine.

### 3.4 Water absorbency test

The water absorption percentage (%) of both hybrid and non-hybrid composites was measured in accordance with ASTM D570-99.^36^ For each composite type, three test specimens were cut to approximately 39 mm in length and 10 mm in width. To prevent water from penetrating, resin was applied to both edges of the specimens and cured. The samples were then placed in an oven at 105 °C for at least one hour to eliminate any remaining moisture. The dry weight of each specimen was recorded using a precision balance, labeled as *W*_i_. Subsequently, the specimens were submerged in water at room temperature (23 °C) for 24 hours. After the immersion period, the samples were gently wiped with tissue paper to remove any surface water and then reweighed, recorded as *W*_f_. The water absorption was calculated using the following formula:1
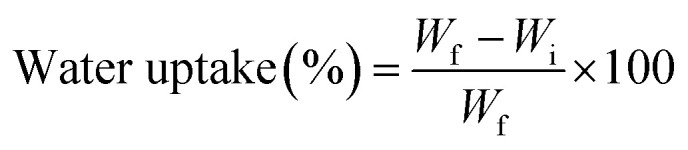


## 4. Results and discussions

### 4.1 Physical properties of wrapped composite

The tables present critical data on the physical and structural properties of jute and glass fibre-reinforced composites. Table 4 highlights the weight distribution and volumetric composition of five composite samples, designated as cJYGF, cGFJY, cJFGF, cGFJF, and G.cGFJF. The weight of the composites ranges from 89.55 g to 105.5 g, with corresponding fibre weight fractions between 58.48% and 65.63%. The volume fractions of fibres range from 52.65% to 60.13%, indicating that fibres dominate the composite structure. G.cGFJF exhibits the highest fibre content and density (1.27 g cm^−3^), suggesting enhanced structural integrity and stiffness. In contrast, cJYGF has the lowest fibre volume fraction (52.65%) and density (1.25 g cm^−3^), implying a relatively lighter and less compact composite. These variations directly affect the mechanical properties and performance of the composites, making fibre volume and weight fractions key design parameters.

Tables 2 and 3 provide insights into the physical characteristics of the preforms used in composite fabrication. Table 2 examines a unidirectional preform made from jute yarn and glass roving. The jute yarn has a direct count of 90 lb per spindle and a linear density of 984 tex, with a fabric thickness of 25 ± 3 mm and GSM of 878 ± 93. The glass roving, with a linear density of 745 tex and twist per inch of 2.68, offers moderate structural reinforcement. Table 3 focuses on a similar preform, using jute fibres instead of yarn, with finer fibre diameters (3.58 ± 0.85 μm) and slightly higher GSM (984 ± 98). Both preforms maintain similar dimensions but differ in structural make-up, influencing fibre alignment and overall composite performance. These findings are significant for optimizing natural fibre-reinforced composites in structural and sustainable material applications.

**Table 2:** The physical properties of jute yarn, glass roving and jute glass wrapped preform

	Jute yarn			Glass roving	Perform thickness (mm)	Preform GSM (g m^−2^)
	Yarn direct count (lb per spindle)	Linear density (tex)	Twist per inch (TPM)	Linear density (tex)		
Unidirectional jute yarn and glass wrapped preform	25(±3)	878(±93)	90(±12)	984(±98)	2.68(±0.25)	745(±84)

**Table 3:** The physical properties of jute fibre, glass roving and jute glass wrapped preform

	Jute fibre		Glass roving	Perform thickness (mm)	Preform GSM (g m^−2^)
	Fibre diameter (μm)	Linear density (tex)	Linear density (tex)		
Unidirectional jute fibre and glass wrapped preform	25(±3)	3.58(±0.85)	984(±98)	2.75(±0.35)	755(±95)

**Table 4:** Physical properties of jute glass wrapped composites

Composite code	Weight of composite, g	Weight of fibres, g	Weight of fibres, g	Weight of composite, g	Weight fraction of fibres, *w*_f_	Weight fraction of matrix, *w*_m_	Volume fraction of fibre, *v*_f_	Volume fraction of matrix, *v*_m_	Density of composite, g cm^−3^
cJYGF	90.5	52.93	52.93	90.5	58.48	41.51	52.65	47.34	1.25
cGFJY	103.33	63.73	63.73	103.33	61.67	38.32	55.89	44.10	1.26
cJFGF	89.55	54.48	54.48	89.55	60.83	39.16	55.12	44.87	1.26
cGFJF	100.3	63.87	63.87	100.3	63.67	36.32	58.05	41.94	1.27
G.cGFJF	105.5	69.24	69.24	105.5	65.63	34.36	60.13	39.86	1.27

### 4.2 Tensile properties

**Fig. 5:**
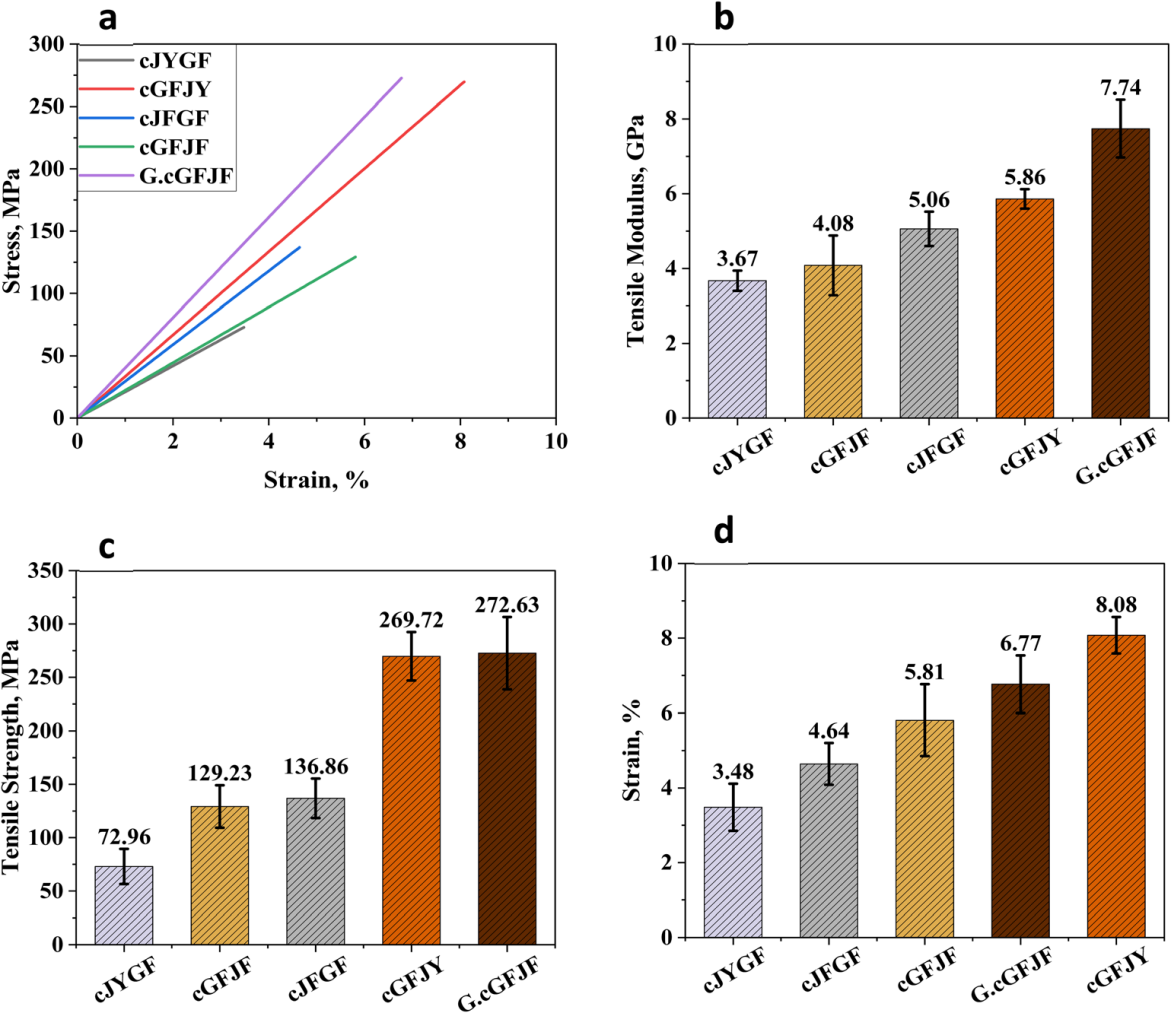
(a) Tensile stress–strain curve, (b) tensile modulus, (c) tensile strength, (d) tensile strain of different wrapped and treated jute–glass hybrid composites.

The mechanical performance of hybrid glass–jute composites is strongly influenced by the choice of core and wrapping fibres and surface modifications such as graphene oxide (GO) treatment, as shown in Fig. 5(a–d). The core glass fibre wrapped with jute yarn (cGFJY) exhibited the highest tensile strength (269.72 MPa), strain (8.08%), and tensile modulus (5.86 GPa) among the untreated composites. This superior performance can be attributed to the high load-bearing capacity of the glass fibre core, which provides enhanced mechanical integrity. While the jute fibre wrapping ensures improved fibre-matrix adhesion. Studies suggest that hybridization of synthetic and natural fibres optimizes stress transfer efficiency to balance strength and toughness in composite structures. The cJYGF composite demonstrated the lowest tensile strength (72.96 MPa), strain (3.48%), and modulus (3.67 GPa). The weaker mechanical performance of this configuration is likely due to the lower strength of jute yarn compared to continuous glass fibres, making the composite more sensible to early failure under tensile loading. Jute yarn is more porous and irregular than unidirectional jute fibre, which affects fibre packing and stress transfer efficiency. Although the glass fibre wrapping provides some reinforcement, it does not sufficiently compensate for the weak jute yarn core. As a result, it reduced tensile properties. The cJFGF composite exhibited moderate mechanical performance, with a tensile strength of 136.86 MPa, strain of 4.64%, and modulus of 5.06 GPa. The higher modulus compared to cJYGF suggests that using a jute fibre core rather than jute yarn contributes to better load distribution and fibre-matrix bonding. The glass fibre wrapping provides additional reinforcement. It improved the overall stiffness and strength. Previous studies indicate that fibre hybridization, particularly when synthetic fibres wrap natural fibres, enhances interfacial adhesion and stress distribution. It reduced the fibre pullout and premature failure. Similarly, the cGFJF composite showed a tensile strength of 129.23 MPa, strain of 5.81%, and modulus of 4.08 GPa. Compared to cGFJY, this composite exhibited lower strength and modulus but higher strain. It indicates that jute wrapping enhances ductility but slightly compromises stiffness. Jute fibres possess high elongation capacity, which contributes to energy absorption. It makes the composite more resistant to sudden failure. The most significant improvement in tensile properties was observed in the GO-treated core glass fibre wrapped with jute fibre (G.cGFJF) composite, which exhibited the highest tensile strength (272.63 MPa), modulus (7.74 GPa), and a strain of 6.77%. The remarkable increase in tensile modulus (∼90% higher than cGFJF) and tensile strength can be attributed to the enhanced interfacial bonding between the fibre and matrix due to the presence of graphene oxide. Studies have shown that GO functionalization improves fibre wettability and roughness. The oxygen-containing functional groups in GO interact strongly with fibre surfaces, facilitating superior load transfer and reducing interfacial voids. As a result, it increases stress transfer efficiency.

### 4.3 Flexural properties

**Fig. 6:**
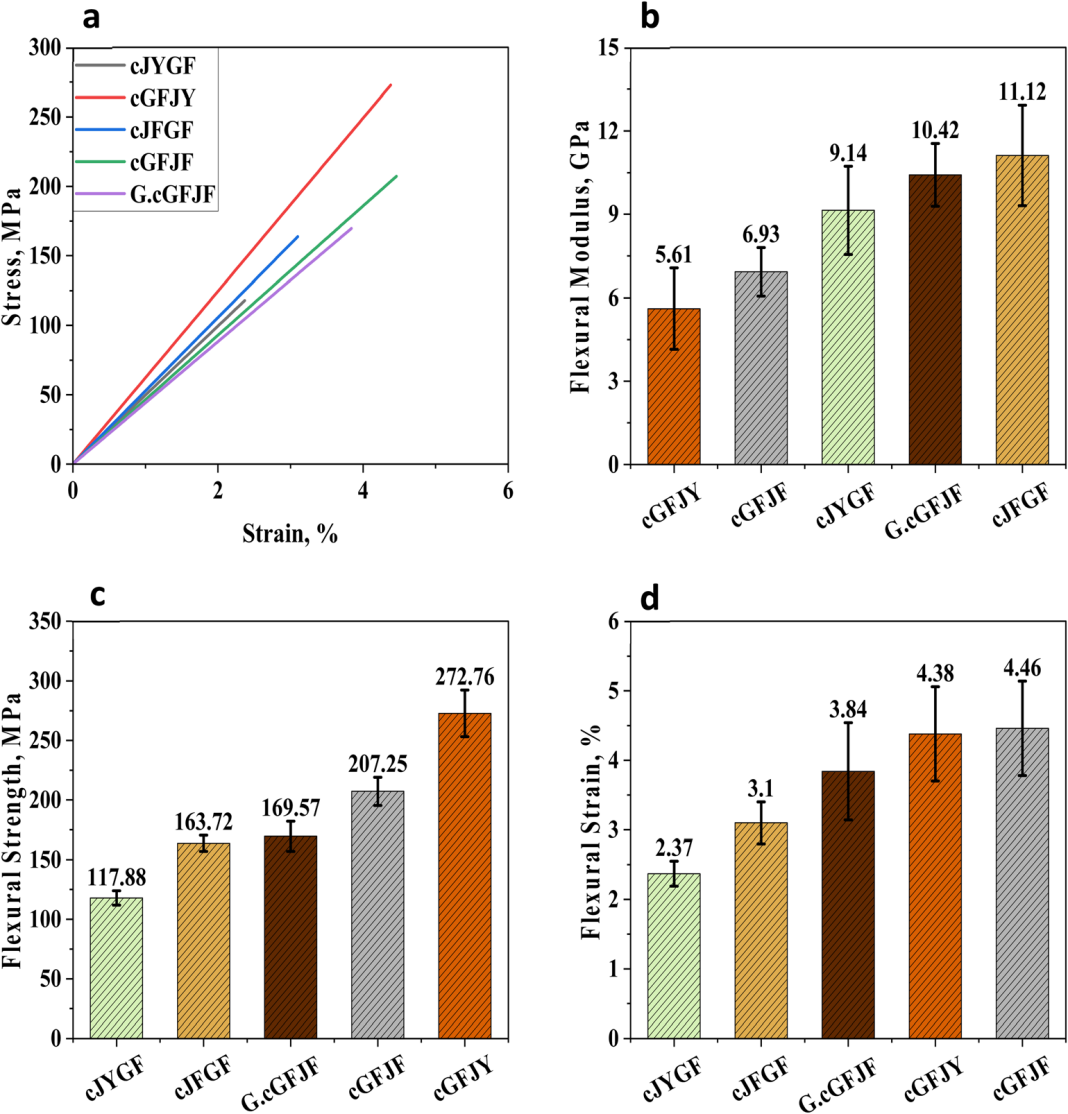
(a) Flexural stress–strain curve, (b) flexural modulus, (c) flexural strength, (d) flexural strain of different wrapped and treated jute–glass hybrid composites.

The flexural properties of hybrid glass–jute composites demonstrate significant variations depending on fibre configuration and surface modifications, shown in Fig. 6(a–d). The results indicate that composites incorporating glass fibre cores generally exhibit superior flexural strength, whereas jute fibre cores contribute to improved stiffness and modulus. Among the untreated composites the core glass fibre wrapped with jute yarn (cGFJY) displayed the highest flexural strength (272.76 MPa) but a relatively lower modulus (5.61 GPa). It suggests strong load-bearing capabilities but limited stiffness due to suboptimal fibre-matrix adhesion. Glass fibres are known to possess high tensile and flexural strength, making them effective reinforcements. Comparatively, the cGFJF composite exhibited a slightly lower flexural strength (207.25 MPa) but a higher modulus (6.93 GPa), indicating improved stress transfer and fibre packing efficiency. The structural composition of jute fibres provides better resistance to deformation, contributing to a stiffer composite. The cJFGF composite exhibited a moderate flexural strength (163.72 MPa) but the highest modulus (11.12 GPa) among the untreated composites. This suggests that the jute fibre core contributes to overall stiffness, while the glass fibre wrapping enhances surface reinforcement. Jute fibres inherently possess high cellulose content, which provides rigidity and structural stability. But their mechanical limitations in terms of strength prevent them from achieving the highest flexural performance. On the other hand, the cJYGF composite exhibited the lowest flexural strength (117.88 MPa) and strain (2.37%), although it maintained a moderate modulus (9.14 GPa). This reduced performance can be attributed to the inherently lower strength of jute yarn compared to jute fibre. The glass fibre wrapping provides external reinforcement but without a strong core the composite lacks the necessary structural integrity to withstand high bending loads. The most notable improvement in mechanical performance was observed in the GO-treated core glass fibre wrapped with jute fibre (G.cGFJF) composite. It exhibited a flexural strength of 169.57 MPa and a significantly improved modulus of 10.42 GPa. The 50% increase in modulus compared to untreated cGFJF indicates that GO treatment enhances fibre-matrix adhesion. It leads to better stress transfer and reduced micro-void formation. Graphene oxide (GO) has been widely recognized for its ability to improve composite properties due to its high surface area, mechanical robustness and strong interfacial interactions with polymer matrices. Studies have shown that GO functionalization enhances fibre wettability, allowing for more efficient load transfer between fibres and the matrix.

### 4.4 Impact strength

**Fig. 7:**
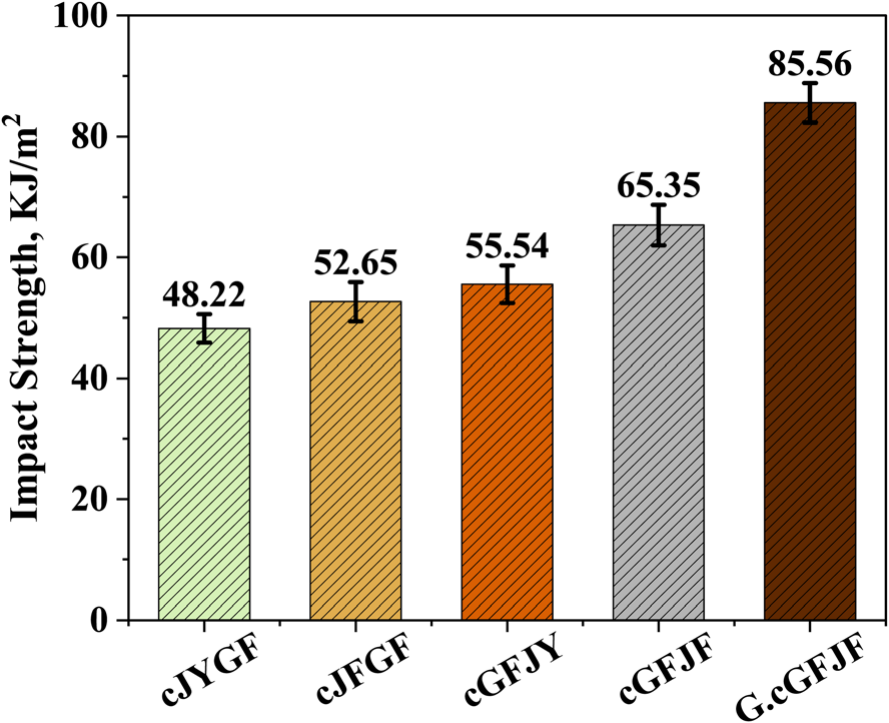
Impact strength of different wrapped and treated jute–glass hybrid composites.

The impact strength analysis of different glass–jute hybrid composites, as shown in Fig. 7. It reveals a significant variation in impact strength based on the core and wrapping fibre arrangements and treatment. Among the five composites, G.cGFJF exhibited the highest impact strength of 85.56 kJ m^−2^. It can be attributed to the GO treatment applied to the fibres. The GO coating enhances fibre-matrix bonding, ensuring better stress transfer and energy dissipation during impact. As a result, it increases the overall toughness of the composite. The cGFJF composite achieved the second-highest impact strength of 65.35 kJ m^−2^. It indicates that glass fibre as the core material significantly improves energy absorption. The cGFJY composite displayed an impact strength of 55.54 kJ m^−2^, slightly lower than cGFJF. It suggests that jute yarn provides a relatively weaker fibre-matrix bonding compared to jute fibre. The cJFGF composite exhibited an impact strength of 52.65 kJ m^−2^. It shows that a jute fibre core does not offer the same level of impact resistance as a glass fibre core due to its lower stiffness and weaker tensile properties. The lowest impact strength of 48.22 kJ m^−2^ was recorded for cJYGF. The reduced strength in this composite can be linked to the lower mechanical properties of jute yarn and potential micro-gaps between the fibre and matrix, which may serve as crack initiation points. The significant improvement in impact strength observed in the G.cGFJF composite highlights the effectiveness of graphene in enhancing the interfacial bonding between glass fibre and the polymer matrix. This study clearly indicates that the core material plays a crucial role in determining impact resistance, with glass fibre cores providing superior performance compared to jute fibre or jute yarn cores. Additionally, surface treatments like GO coating further enhance mechanical properties, making G.cGFJF the best-performing composite.

### 4.5 Water absorbency test

**Fig. 8:**
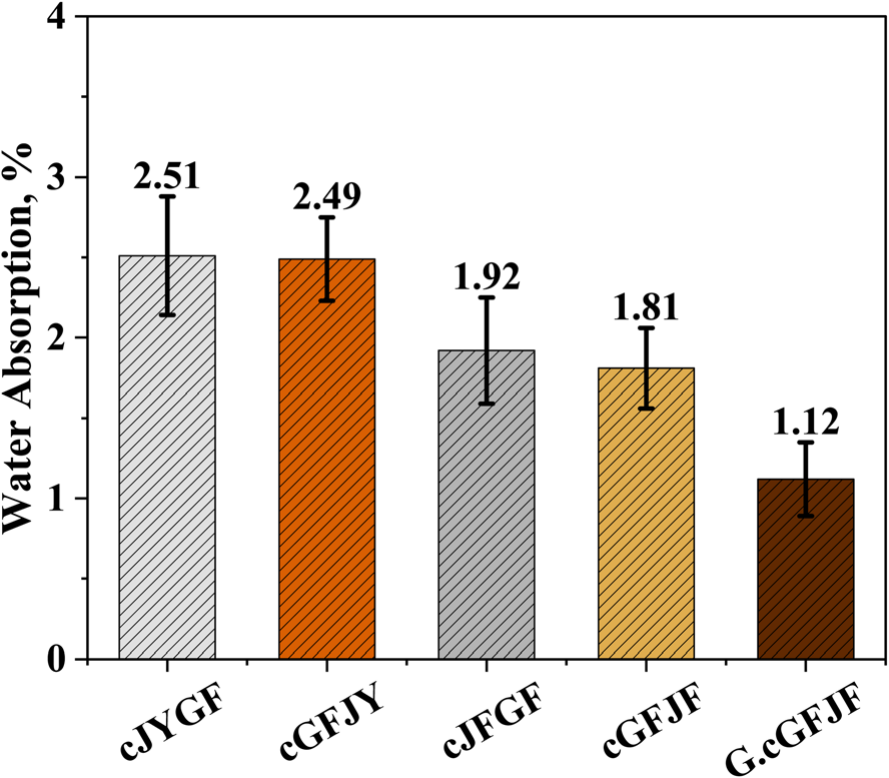
Water absorbency of different wrapped and treated jute–glass hybrid composites.

A significant drawback of lignocellulosic fibres, such as jute, is their tendency to absorb water, leading to dimensional instability and mechanical property degradation. The hydroxyl groups present in lignocellulosic fibres readily attract moisture, causing fibre swelling and shrinkage upon drying. The results (Fig. 8) indicate that the composite with a cJYGF composite exhibited the highest water absorption (2.51%), followed closely by the composite cGFJY composite at 2.49%. These values demonstrate the hydrophilic nature of jute, which allows for higher moisture uptake when incorporated as the core or outer layer. The composite cJFGF composite had a reduced water absorption of 1.92%, indicating a partial barrier effect provided by the outer glass fibre layer. Further reduction in water absorption was observed in the composite with a glass fibre core and a jute fibre wrapping (cGFJF) which absorbed 1.81% water. This suggests that using glass fibre as the core provides better resistance to moisture uptake than using jute. The most significant reduction in water absorption was observed in the GO-treated composite (G.cGFJF) which exhibited the lowest water absorption of 1.12%. This decrease can be attributed to the graphene oxide (GO) treatment which likely enhanced the hydrophobic nature of the fibre-matrix interface by reducing the availability of hydroxyl groups for water absorption.

### 4.6 Fracture morphology of the composites

Fracture specimens obtained after tensile testing were physically examined to understand the failure mechanisms of hybrid composites fabricated using different roving combinations of field-retted jute fibers, commercial jute yarn, and glass fibers. Representative digital images of the fractured composites are presented in Fig. 4. It was observed that the hybrid composites comprising both jute and glass fibers did not exhibit complete failure during tensile testing. This behavior is attributed to the mismatch in stress distribution between the jute and glass fibers, where the mechanically weaker jute fibers fractured first while the glass fibers remained intact but exhibited splitting behavior.

**Fig. 9:**
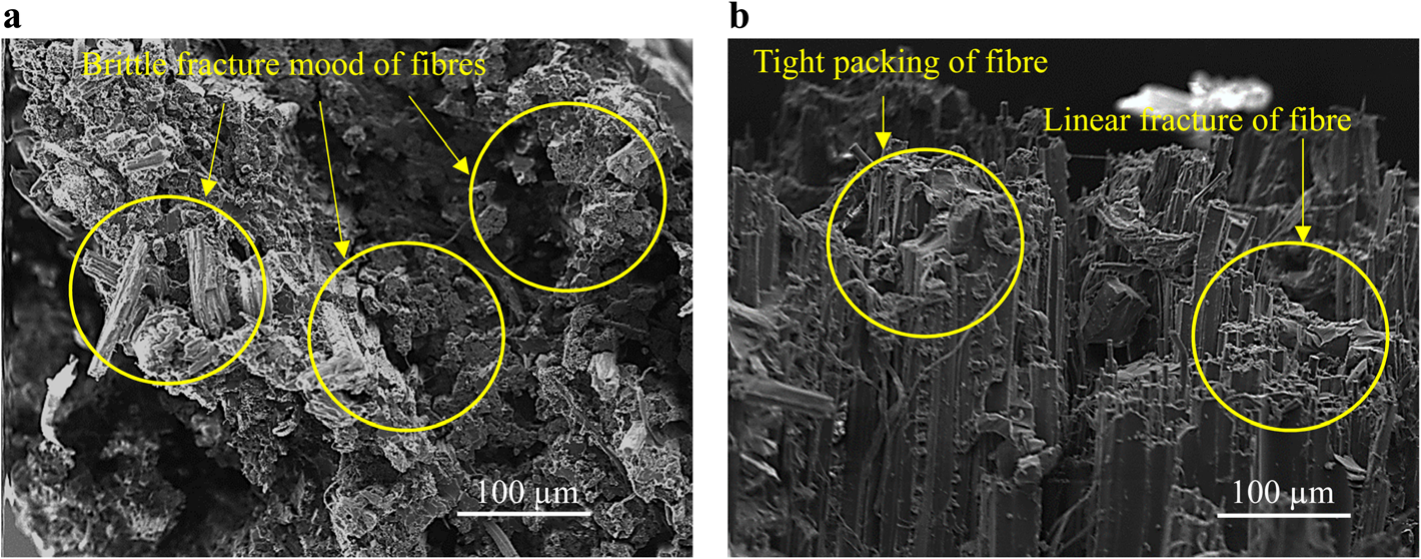
(a) SEM fracture specimen of filed retted jute roving composite without GO coating at 250× magnification, (b) SEM fracture specimen of field retted jute roving composites with GO coatings at 250× magnification.

Among the tested systems, the two best-performing hybrid configurations—cGFJF and GcGFJF—were selected for detailed investigation. Since the glass fibers did not fail completely, only the fractured regions containing jute fibers (with and without graphene oxide (GO) treatment) were analyzed. The corresponding scanning electron microscopy (SEM) images of the fracture surfaces are shown in Fig. 9(a and b). In the untreated composites, irregular and brittle fracture morphology was observed, with a higher incidence of fiber pull-out. This behavior is likely due to the presence of surface impurities such as hemicellulose, lignin, and waxes, which impede effective interfacial adhesion and create stress concentration zones, resulting in uneven fiber failure. In contrast, GO-treated jute fibers exhibited significantly improved interfacial bonding. The alkali treatment removed hemicellulose, exposing more hydroxyl groups on the fiber surface, which interacted strongly with the GO solution through hydrogen bonding. During composite fabrication, these GO-functionalized fibers formed robust chemical bonds with the epoxy matrix, particularly *via* amide/peptide linkages with the amine hardener. This chemical affinity enhanced fiber packing density and promoted uniform stress distribution under tensile loading, leading to a more homogeneous fiber breakage pattern, as shown in Fig. 9b.

## 5. Conclusion

In this study, unidirectional (UD) hybrid fibre-reinforced polymer composites were successfully developed using individualized jute fibres and glass fibres through an innovative wrapping technique that formed core-sheath preforms. These preforms were coated with graphene oxide (GO) to improve interfacial adhesion and then fabricated into composites *via* compression molding. Five composite configurations were investigated—cJYGF, cGFJY, cJFGF, cGFJF, and G.cGFJF—with variations in the core-wrapping arrangement and surface treatment. The performance of each composite was evaluated through tensile, flexural, impact, and water absorption tests to determine their suitability for structural applications.

From the experimental results, several key findings emerged regarding the influence of fibre architecture, material placement, and GO surface modification:

• The GO-treated composite G.cGFJF, in which glass fibre was used as the core and jute fibre as the wrapping, demonstrated the highest overall mechanical performance. It achieved the maximum tensile strength, tensile modulus, and impact strength showing the critical role of graphene oxide in enhancing fibre-matrix interaction and stress transfer. This configuration outperformed all untreated counterparts, confirming the effectiveness of surface engineering in hybrid composites.

• Among the non-treated composites, cGFJY (glass core, jute yarn wrap) showed the highest tensile and flexural strengths. This superior performance is attributed to the high load-bearing capability of the glass core and the ductility offered by the jute yarn shell. Meanwhile, the cJFGF configuration exhibited the highest flexural modulus indicating the significant influence of jute fibre alignment in the core on composite stiffness.

• Water absorption studies showed that natural fibre-rich composites absorbed more moisture, with cJYGF showing the highest uptake (2.51%). However, the G.cGFJF composite recorded the lowest absorption rate (1.12%) due to the barrier effect of glass in the core and the hydrophobic nature of the GO coating. This water resistance improves the dimensional stability and longevity of the hybrid system, which is essential for real-world structural applications.

• The hybrid architecture with a core-sheath configuration and UD alignment enabled superior stress distribution, improved packing density, and greater fibre-matrix interaction. These factors collectively contributed to enhanced mechanical and environmental properties of the composites.

The research validates the concept that natural/synthetic hybridization, particularly using jute fibre and glass fibre in a UD pattern with GO surface treatment, can produce composites that are not only mechanically strong but also environmentally responsible. The developed G.cGFJF configuration stands out as a promising candidate for next-generation lightweight structural materials in automotive, civil, and aerospace sectors. Its potential to reduce synthetic fibre content while maintaining structural integrity marks a significant advancement in sustainable composite engineering.

## Author contributions

Forkan Sarker contributed to conceptualization, supervision, experimental design, data analysis, and review and editing. Mainul Islam contributed to experimental design, composite manufacturing, testing, data analysis, writing the original draft, and editing. Md Tarikul Islam contributed to experimental design, composite manufacturing, and testing. Main Uddin Apu contributed to experimental design, composite manufacturing, and testing. Ariful Islam contributed to experimental design, composite manufacturing, and testing. Shahjalal Khandaker analyzed data and reviewed the manuscript.

## Conflicts of interest

There are no conflicts to declare.

## Data Availability

The data that support the findings of this study are available from the corresponding author upon reasonable request.
